# Large Cell Neuroendocrine Carcinoma Presenting as Adult Intussusception

**DOI:** 10.7759/cureus.51546

**Published:** 2024-01-02

**Authors:** John O Agboola, Hagar Attia, Li Zhonghua, Meredith Pittman

**Affiliations:** 1 Pathology Anatomic & Clinical, State University of New York (SUNY) Downstate Health Science University, Brooklyn, USA; 2 Pathology, Kings County Hospital Center, Brooklyn, USA; 3 Pathology, Kings County Hospital Center, New York, USA; 4 Department of Pathology, Maimonides Medical Center, Brooklyn, USA

**Keywords:** large-cell neuroendocrine carcinoma, gastrointestinal neuroendocrine tumor, adult intussusception, colonic adenocarcinoma, right hemicolectomy, colon resection, ileo-colic intussusception, large cell neuroendocrine

## Abstract

Large cell neuroendocrine carcinoma (LCNEC) is an extremely rare malignant tumor of the colon, presenting with more severe clinical outcomes in comparison to colonic adenocarcinoma. There are very few reported cases in the literature. We hereby add our voice to the incidence of this disease by presenting the first report of a patient with ileocolic intussusception secondary to a large cell neuroendocrine cancer of the cecum. The patient was a 48-year-old woman who presented with acute onset of generalized abdominal pain and leukocytosis. CT scan revealed an ileocecal intussusception and multiple liver metastases suggestive of a malignant bowel lesion. She underwent emergency surgery, and an extended right hemicolectomy with ileo-transverse anastomosis was performed. Histology of the resected lesion revealed large cell neuroendocrine carcinoma of the cecum with invasion through the muscularis propria into peri colorectal tissues. The tumor retained mismatch repair (MMR) proteins with low potential for microsatellite instability (MSI). With a clinical diagnosis of stage IV LCNEC, the patient began platinum doublet chemotherapy with carboplatin and etoposide; however, her disease progressed, and the patient expired within a few months after her diagnosis. Clinical diagnosis of adult intussusception should prompt clinicians to rule out malignant etiology. This patient had a large cell neuroendocrine carcinoma of the colon, a rare and extremely aggressive malignancy. Patients with LCNEC will benefit from a multidisciplinary approach to treatment.

## Introduction

Large cell neuroendocrine carcinoma accounts for only 0.2-0.3% of all malignant tumors of the colon and is associated with worse clinical outcomes when compared to colonic adenocarcinoma. They are much rarer than neuroendocrine tumors associated with other organs, such as the lung. However, patients with this disorder are discovered late and are sometimes diagnosed on evaluation for acute gastrointestinal symptoms, and it comes with a worse outcome compared to adenocarcinoma of the colon. Because it is not a common clinical entity, there are very few reported cases in literature and most of these are of patients in their seventh or eighth decade. We hereby add our voice to the incidence of this disease in a younger female patient who presents as an emergency to our center. This will be the first report in the literature of a patient presenting with ileocolic intussusception secondary to a lead point formed by a large cell neuroendocrine cancer of the cecum. Also, this patient is much younger than the usual age group of patients with similar diagnoses. Awareness of this disease and prompt reporting of cases will encourage more research and clinical trials and hopefully help find treatment with better outcomes.

## Case presentation

**Figure 1:**
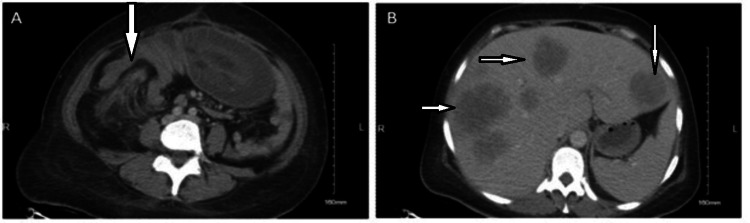
CT scan images of the patient A. CT scan image showing the ileocolic intussusception (arrow) in the 48-year-old patient with Large Cell Neuroendocrine Carcinoma (LCNEC). B. The patient had multiple lesions in the liver (arrows) at presentation suggestive of hepatic metastasis.

A 48-year-old woman presented to the emergency department with acute onset of generalized abdominal pain. She had a history of previous uterine myomectomy, but no other significant past medical history. On physical exam, she was found to be tachycardic but afebrile with a soft abdomen, and she stated that she was passing flatus and having bowel movements. Laboratory work-up was remarkable for a leukocytosis of 30K/ul (reference range 3.5-9.6 10^9^/L) [1]. CT scan revealed both an ileocolic intussusception (Figure 1A) and several hypodense masses in the liver suggestive of metastasis (Figure 1B). The patient underwent emergency surgery, which confirmed the intussuscepted colon and multinodular liver with palpable masses. Because the intussusception could not be reduced intra-operatively, the patient had an extended right hemicolectomy with ileo-transverse anastomosis.

**Figure 2:**
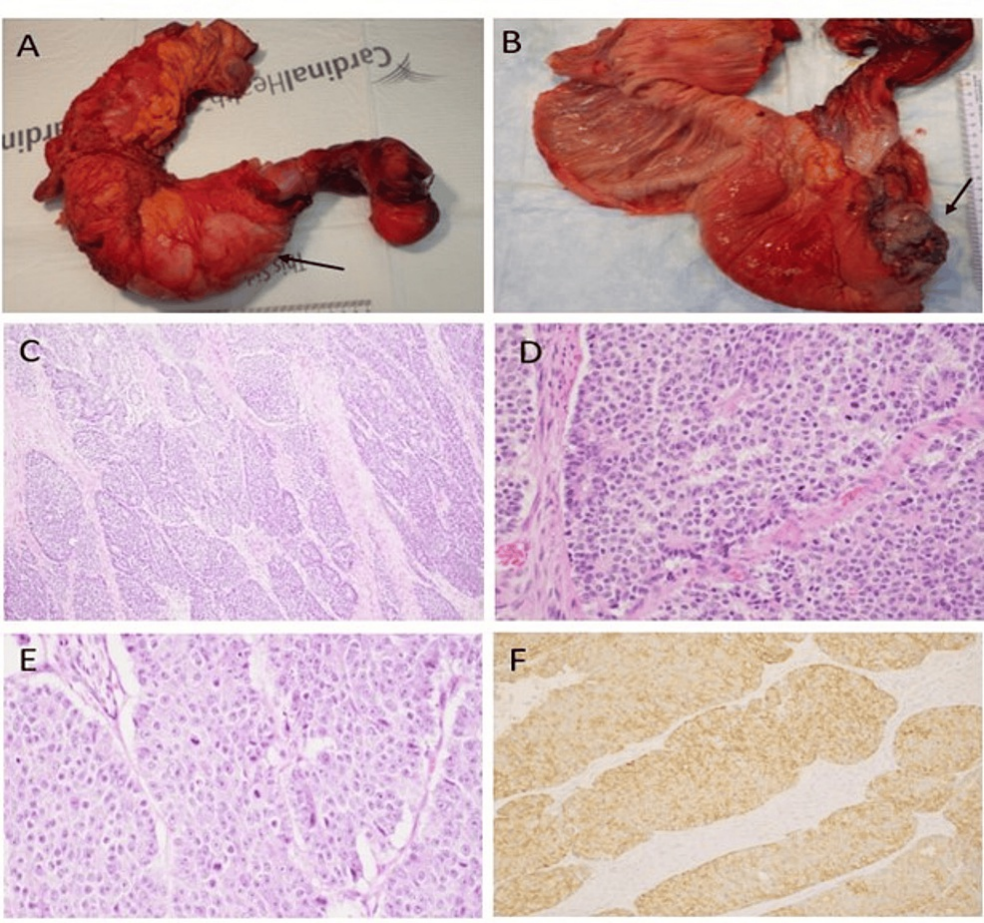
Gross examination and histopathology images (A, B). Gross images show ileocolic intussusception (black arrow) opened to reveal a fungating ulcerated cecal pole tumor. The serosa and the appendix are not involved. (C, D). Organoid and pseudo glandular patterns of neoplastic cells. E. Large cells with pleomorphism, moderate to abundant eosinophilic cytoplasm, and prominent nucleoli F. Synaptophysin immunohistochemistry (IHC).

On gross examination, the intussuscepted region was opened to show an 8 x 5 x 3 cm ulcerated mass in the cecum with hyperemia and edema of the proximal ascending colon (Figures 2A, 2B). Histologic review of the cecal mass showed sheets of large neoplastic cells with moderate to abundant granular cytoplasm and a brisk mitotic rate. Atypical mitotic figures were noted. The malignant cells were arranged in organoid-like forms with central necrosis, and focal rosette-like, and trabecular growth patterns were identified, as well (Figures 2C-2E). The tumor invaded through the muscularis propria into pericolorectal tissues, and two of 16 lymph nodes were positive for metastatic disease. Immunohistochemical stains were performed, and the cells co-expressed chromogranin and synaptophysin (Figure 2F) with a Ki-67 proliferative index of 60-70%.

Overall, the morphologic and immunophenotypic features were those of a large cell neuroendocrine carcinoma (LCNEC) of the cecum. The tumor retained mismatch repair (MMR) proteins with low potential for microsatellite instability (MSI). Because of the liver metastases noted intraoperatively, the patient was clinically staged as a cT3 N1b M1. 

The patient’s postoperative course was complicated by anasarca, persistent anemia, fever, and tachycardia. A repeat CT scan two months after surgery revealed the progression of hepatic metastasis and possible pulmonary metastases. Although she began platinum doublet chemotherapy with carboplatin and etoposide, there was a further progression of the disease, and the patient died within four months of her diagnosis.

## Discussion

Intussusception occurs when a proximal segment of the bowel, the intussusceptum, telescopes into a distal segment of the bowel, the intussuscipiens. Most (95%) cases occur in children younger than two years who present with the classic triad of colicky abdominal pain, bloody mucoid diarrhea, and vomiting. A diagnosis of intussusception is more difficult to make in adults who may present with nonspecific symptoms, such as the generalized abdominal pain of our patient. Imaging studies are crucial for timely diagnosis. Both Ultrasound, preferred for children, and CT show a characteristic double lumen or donut sign of bowel within the bowel. The most common etiologies for intussusception in adults are fibrous adhesions secondary to prior abdominal surgery and malignancy, the latter of which is the underlying cause in over 50% of cases in some adult intussusception series [2,3]. Intussusception also accounts for up to 5% of intestinal obstruction in adults, and surgical resection of the affected segment is considered the definitive treatment of choice [3]. For our patient, resection was necessary after unsuccessful intraoperative reduction. To our knowledge, this is the first description of a large cell neuroendocrine cancer of the cecum presenting as intussusception. 

Large cell neuroendocrine carcinoma (LCNEC) is defined as a poorly differentiated or high-grade carcinoma with large cell features that expresses at least one “neuroendocrine” marker of differentiation [4]. The incidence of all colorectal cancers, which are predominantly adenocarcinoma, is estimated to be 32.5 per 100,000 per year [5]. In contrast, colorectal neuroendocrine carcinoma (NEC), small or large cell type, is extremely rare with an incidence of only 1-2 per million per year [6]. On gross and histologic examination, neuroendocrine carcinoma may look similar to the much more common adenocarcinoma. Histologically, features that raise the possibility of LCNEC include an organoid pattern or growth, absence of true glands, and immunohistochemistry (IHC) staining for neuroendocrine markers such as synaptophysin, chromogranin, Neuro-specific Enolase (NSE), and CD56.

Although LCNEC can occur in any organ, the most common site of involvement is the lung. Pulmonary LCNEC is strongly linked to smoking, and most patients present at advanced stages of the disease with poor survival outcomes [7]. Similarly, patients with colorectal LCNEC usually present with high-stage disease and a corresponding poor prognosis [8-11]. A single institution report of 38 patients with colorectal NEC diagnosed over 22 years found the average age of diagnosis to be 57 years with a median survival of only 10.4 months and most of the patients (69%) had metastatic disease at the time of presentation [9].

There is currently no standard regimen or treatment specific for large cell neuroendocrine cancer of the colon. Many treatment regimens used for LCNEC of the colon are adapted from a therapeutic analysis of patients with pulmonary high-grade NECs [12]​. Corbett et al. (2021) noted three chemotherapeutic regimens, FOLFOX, FOLFIRI, and CAPTEM, that can be used for non-pulmonary LCNEC​ [13]. Microsatellite instability is more common in colonic NEC than colonic adenocarcinoma (up to 30% in some series), and targeted therapy can be used in these patients [14]. For high-grade neuroendocrine tumors, the North American Neuroendocrine Tumor Society (NANETS) recommends surgical resection of the primary tumor, embolectomy for liver metastasis, and chemotherapy for patients with advanced diseases [12]​. Our patient's tumor was mismatch repair proficient and metastatic at the time of presentation. We attempted to enroll her in a clinical trial, but none was available. After resection of the primary, she started on carboplatin/etoposide therapy, but she received only the first dose before succumbing to the disease.

## Conclusions

The finding of adult intussusception should prompt clinicians to rule out a malignant etiology. In this case, the culprit was a large cell neuroendocrine carcinoma of the colon, a rare and highly aggressive malignancy. Although LCNEC may mimic colonic adenocarcinoma histologically, the presence of solid and organoid growth and an absence of glands should prompt staining for neuroendocrine markers. Because these patients often present with high-stage disease, they benefit from a multidisciplinary approach to treatment, although there is a need for further research to evaluate the best and safest form of adjuvant treatment that will improve clinical outcomes in these patients.
